# Exploration of the mechanism of chemobrain related to neuroinflammation from 1994 to 2023

**DOI:** 10.1016/j.clinsp.2026.100924

**Published:** 2026-03-28

**Authors:** Hansong Yu, Yanting Zhou, Xiaoxiao Wang, Yuetong Pan, Hongyan Li

**Affiliations:** General Surgery Department, Xuanwu Hospital, Capital Medical University, Beijing, China

**Keywords:** Chemotherapy, Neuroinflammation, Chemobrain, Cognitive dysfunction, Cytokines

## Abstract

**Background:**

“Chemobrain”, or chemotherapy-related cognitive impairment, is a well-documented clinical phenomenon. While neuroinflammation has been proposed as a key underlying mechanism, a comprehensive mapping of the knowledge structure and evolution of this specific research domain is lacking.

**Methods:**

The authors conducted a bibliometric analysis to quantitatively and visually characterize the scholarly literature on neuroinflammation and chemobrain. A total of 923 relevant publications from the Web of Science Core Collection (1994‒2023) were analyzed using VOSviewer.

**Results:**

The analysis reveals a rapidly growing field, with a significant increase in publications and thematic focus on terms like cytokines, microglia, and oxidative stress in recent years. This shift signifies a move from purely descriptive studies toward mechanistic inquiry. While the United States has historically been the dominant contributor, China has emerged as a leading force in the past decade, indicating a global expansion of research interest.

**Conclusion:**

This study provides the first holistic overview of the neuroinflammation and chemobrain research landscape, delineating its thematic evolution and collaborative networks. The findings underscore that neuroinflammation remains a highly active, yet not fully elucidated, mechanistic hypothesis driving the field. A key limitation is the bibliometric nature of the study, which reflects terminological and publication trends rather than direct biological validation. Future research should focus on integrating multi-omics data and conducting longitudinal clinical studies to substantiate these mechanistic links.

## Highlights

• First 30-year bibliometric map of Chemobrain-neuroinflammation research.
• Neuroinflammation is the central, sustained mechanistic focus of the field.
• Microglia, BBB, oxidative stress, and gut-brain axis are key research hotspots.
• Multi-omics and clinical trials are urgently needed for translational breakthroughs.

## Introduction

In 2020, approximately 19.3 million new cancer cases were diagnosed globally, with nearly 10.0 million deaths attributed to the disease.^1^ Chemotherapy is an effective treatment for cancer, but it also has certain side effects, including gastrointestinal reaction, bone marrow suppression, neurotoxicity and so on. As a widely used therapeutic approach, chemotherapy is effective but accompanied by various adverse effects. Among these, Chemotherapy-Induced Cognitive Impairment (CICI), commonly known as “chemobrain”, has gained increasing attention. CICI is characterized by impairments in cognitive domains such as short-term memory, attention, and executive function, affecting up to 70 % of patients with non-central nervous system cancers.^2^ Up to 70 % of patients with non-central nervous system cancers develop cognitive impairment during or after chemotherapy.^3^ A deeper understanding of chemobrain has become a clinically urgent issue.

The pathogenesis of CICI is multifactorial, involving neuroinflammation, oxidative stress, and direct or indirect neurotoxicity induced by chemotherapeutic agents.^4^^,^^5^ Notably, even chemotherapeutic drugs with poor blood-brain barrier permeability can trigger systemic inflammatory responses, leading to neuroinflammation and cognitive decline.^6^^,^^7^ Emerging evidence also highlights the role of the gut–brain axis, wherein chemotherapy-induced gut microbiota dysbiosis and barrier disruption activate microglia and sustain neuroinflammatory states.^8^^,^^9^ For instance, a recent study demonstrated that methotrexate induces persistent microglial activation, which in turn triggers astrocyte reactivity, contributing to sustained neuroinflammation and neuronal dysfunction.^8^ Additionally, anthracyclines such as doxorubicin promote oxidative stress and mitochondrial dysfunction, leading to lipid peroxidation and indirect neurotoxicity despite limited Blood-Brain Barrier (BBB) penetration.^10^ Platinum-based agents cause DNA crosslinking and provoke neuroinflammation both peripherally and centrally, while taxanes disrupt microtubule dynamics in neurons, exacerbating cognitive deficits.^11^^,^^12^ The treatment options for chemotherapy-induced cognitive impairment can be broadly categorized into pharmacological and non-pharmacological approaches, many of which target pathways related to neuroinflammation and oxidative stress. Despite these advances, the specific mechanisms of neuroinflammation in chemobrain warrant further systematic investigation.

Although substantial scholarly attention has been directed toward both chemobrain and neuroinflammation as discrete research areas, the intellectual convergence and interdisciplinary connections between these fields remain inadequately characterized. Bibliometric approaches provide a rigorous framework for mapping such complex scholarly landscapes through the systematic examination of publication dynamics, conceptual architectures, and collaborative networks across temporal and spatial dimensions. Such methodology proves particularly valuable for identifying established research fronts, detecting emerging thematic concentrations, and revealing structural gaps that might escape detection through conventional review methodologies. Therefore, the authors conducted a 30-year bibliometric analysis (1994‒2023) with the following objectives: 1) To identify key thematic trends and knowledge gaps in chemobrain and neuroinflammation research, 2) To evaluate the contributions and collaborations among authors, institutions, and countries across three decades, and 3) To map the evolution of research hotspots and emerging topics, thereby providing a foundation for guiding future research directions and resource allocation in this field. The findings are expected to inform strategic planning for mechanistic studies, intervention development, and translational research in chemobrain and neuroinflammation.

## Methods

### Source of data and search methodology

This bibliometric investigation delved into articles pertaining to neuroinflammation, cancer, chemotherapy, and chemobrain, spanning the period from 1994 to 2023. To identify temporal trends, the study period was divided into three decades (1994‒2003, 2004‒2013, and 2014‒2023). The Web of Science online database (Clarivate Analytics, Philadelphia, PA, USA) was accessed on August 6, 2024. Two different sets of subject searches were conducted using the advanced search option. The “TS” designation indicates a subject search that retrieves results related to the title, abstract, and keywords of publications. The complete search strings for each set are outlined below:

Set 1 (Neuroinflammation): TS = (Tumor necrosis factor-alpha or Interferon or IL or Macrophage or Inflammatory cytokine or Interleukin or Pro-inflammatory cytokine or CRP or IFN or Cytokine or Chemokine or TNF or Lymphocyte or Microglia or Inflammatory factor or C-reactive protein or Transforming growth factor or TGF)

Set 2 (Chemobrain): TS = (chemobrain OR chemofog OR (cognitive dysfunction AND cancer) OR (cognitive impairment AND cancer))

Each set included the following indexes: WoSCC with a Timespan spanning from 1994 to 2023. The fusion of the two sets was accomplished using the ‘AND’ Boolean operator. The following document types were excluded: meeting abstracts, conference proceedings, book chapters, and retracted publications. Duplicate records were removed. Data extraction involved downloading comprehensive records and cited references in tab-delimited text files. The data, comprising “full records and cited references”, encompassed details such as publication title, publication year, abstract, authorship, author keywords, journal title, citation count, institution, and country.

### Data analyses and presentation

Data were imported into VOSviewer version 1.6.20 (Centre for Science and Technology Studies, Leiden University, Leiden, The Netherlands) for bibliometric analysis.^13^ Term maps were generated using the following options/commands: “Create a map based on bibliographic data”, “Read data from bibliographic database files”, “Type of analysis: Co-occurrence”, “Unit of analysis: All keywords”, and “Counting method: Full counting”. To refine the analysis as previously described, a thesaurus file was crafted using the top 5000 common words from the Corpus of Contemporary American English.^14^^,^^15^ Additional general terms were included in the thesaurus to exclude terms such as “method” and “result”, while terms pertaining to cognition and brain were omitted to facilitate their inclusion in the analysis. The thesaurus was further expanded to ensure that VOSviewer software could recognize terms such as “cognitive impairment”, “cognitive dysfunction”, and “chemobrain” as synonymous.

The software utilizes co-occurrence analysis to extract keywords, where the relatedness of terms is depicted by their co-occurrence frequencies. Thresholds are implemented to sift through all keywords and refine the results. Default thresholds denote the minimum occurrence of a keyword within a document. For each period, the minimum keyword occurrence thresholds were set as follows: 1994∼2003 ‒ 4 ×; 2004∼2013 ‒ 7 ×; 2014∼2023 ‒ 15 × . This adjustment accounts for the increased occurrence of keywords due to more publications incorporating terms related to brain and cognition. To visualize the average number of citations received by documents where a term appears, the parameter of “averaged citations” was utilized. The VOSviewer software constructs a term map based on co-occurrence frequencies. To provide an overview of the field's overall status, a network and density visualization of keywords from 1994 to 2023 was generated using the VOSviewer software, with a minimum keyword occurrence set at least 15.

Publication and citation data for the period spanning 1994 to 2023 were meticulously extracted on August 6, 2024. In conducting the citation analysis, a comprehensive dataset of 49,532 citations, encompassing both other-citations and self-citations, was employed. To visually represent the geographical distribution of countries in relation to the analyzed papers, a detailed map was generated utilizing the 3D Map feature within Excel 2019, a product of Microsoft headquartered in Redmond, WA, USA.

This systematic review and bibliometric analysis were conducted and reported in accordance with the PRISMA (Preferred Reporting Items for Systematic Reviews and Meta-Analyses) guidelines.

## Results

### Publication output trends (1994–2023)

**Fig. 1:**
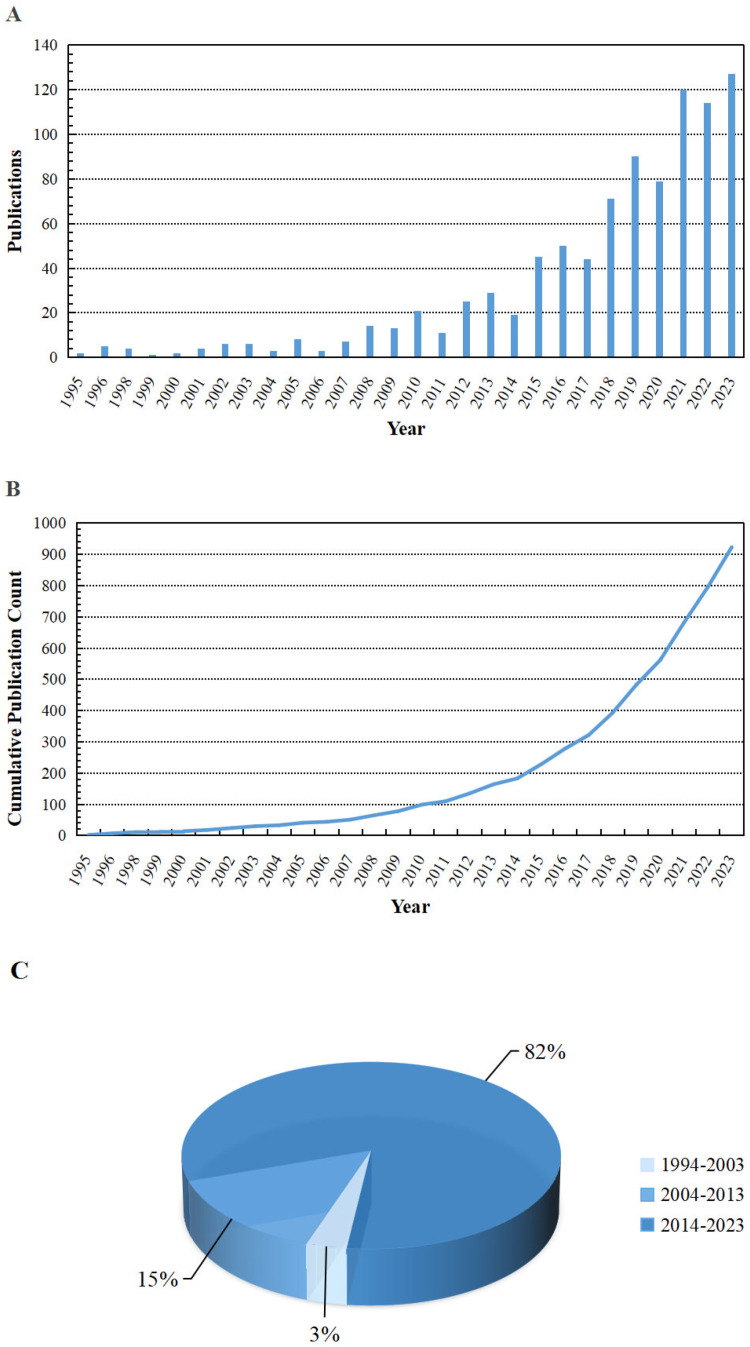
**Publications on neuroinflammation and chemobrain from 1994 to 2023.** (A) Number of publications about neuroinflammation and chemobrain per year. (B) Cumulative number of publications about neuroinflammation and chemobrain per year. (C) Percentage of publications on neuroinflammation and chemobrain per decade.

The bibliometric analysis identified a total of 923 publications focusing on neuroinflammation and chemobrain, revealing a remarkable evolution in research activity over the three decades. The field was in its nascent stage from 1994 to 2003, with a modest output of only 30 publications. A period of steady growth followed between 2004 and 2013, yielding 134 publications and signaling a growing recognition of the link between chemotherapy and cognitive impairment. The most dramatic expansion occurred from 2014 to 2023, during which research output surged to 759 publications. This exponential growth is further highlighted by key milestones: the cumulative number of publications surpassed 100 in 2010, exceeded 500 by 2020, and has consistently seen over 100 articles published annually since 2021 (Fig. 1A–C). This trajectory underscores the field's transition from a niche interest to a major focus of oncological and neurological research, reflecting the increasing clinical concern for the long-term quality of life of cancer survivors. The accelerating trend suggests that chemobrain is now widely recognized as a significant clinical challenge, warranting comprehensive mechanistic and therapeutic investigations.

### Evolving research foci: term co-occurrence and cancer types

**Fig. 2:**
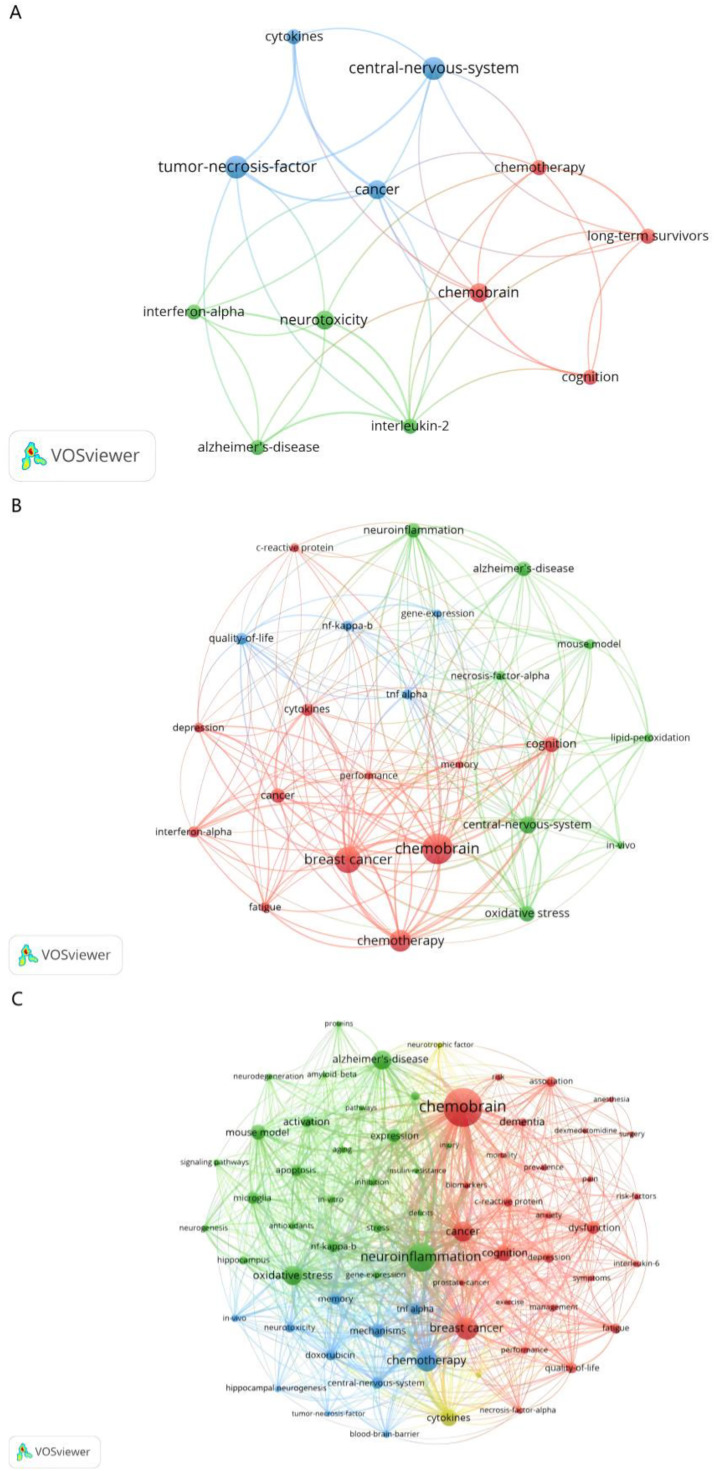
**Term map for every 10-years from 1994 to 2023.** (A) Term map for 1994‒2003. This map shows the visualization of 12-terms occurred at least 4 times in publications from 1994 to 2003. (B) Term map for 2004‒2013. This map shows the visualization of 24 terms occurred at least 7 times in publications from 2004 to 2013. (C) Term map for 2014‒2023. This map shows the visualization of 68 terms occurred at least 15 times in publications from 2014 to 2023. The map shows different terms as circles. The bigger the circle, the more often the term appears. The color scale shows the average citation count for each term.

The shifting priorities within the field are clearly mapped through the evolution of key terminology, which reveals both the consolidation of core concepts and the emergence of new research frontiers. The initial decade (1994‒2003) was characterized by foundational concepts, with term co-occurrence centered on broad themes like “neurotoxicity”, “cancer”, and the newly identified condition of “chemobrain” (Fig. 2A). This pattern suggests that early research primarily focused on establishing the clinical phenomenon itself, with limited exploration of underlying biological mechanisms.

The subsequent decade (2004‒2013) witnessed a significant expansion in scope, as research began to delve into underlying mechanisms. This was evidenced by the emergence of highly cited terms such as “C-reactive protein”, “NF-Κb”, and “tumor necrosis factor-α”, pointing toward an investigative focus on inflammatory pathways. During this period, the strong linkage between “breast cancer”, “chemobrain”, and “neuroinflammation” firmly established breast cancer as the predominant disease model for studying this phenomenon (Fig. 2B), a trend that has profoundly shaped the landscape of the field but also highlights a potential research gap regarding other cancer types.

**Fig. 3:**
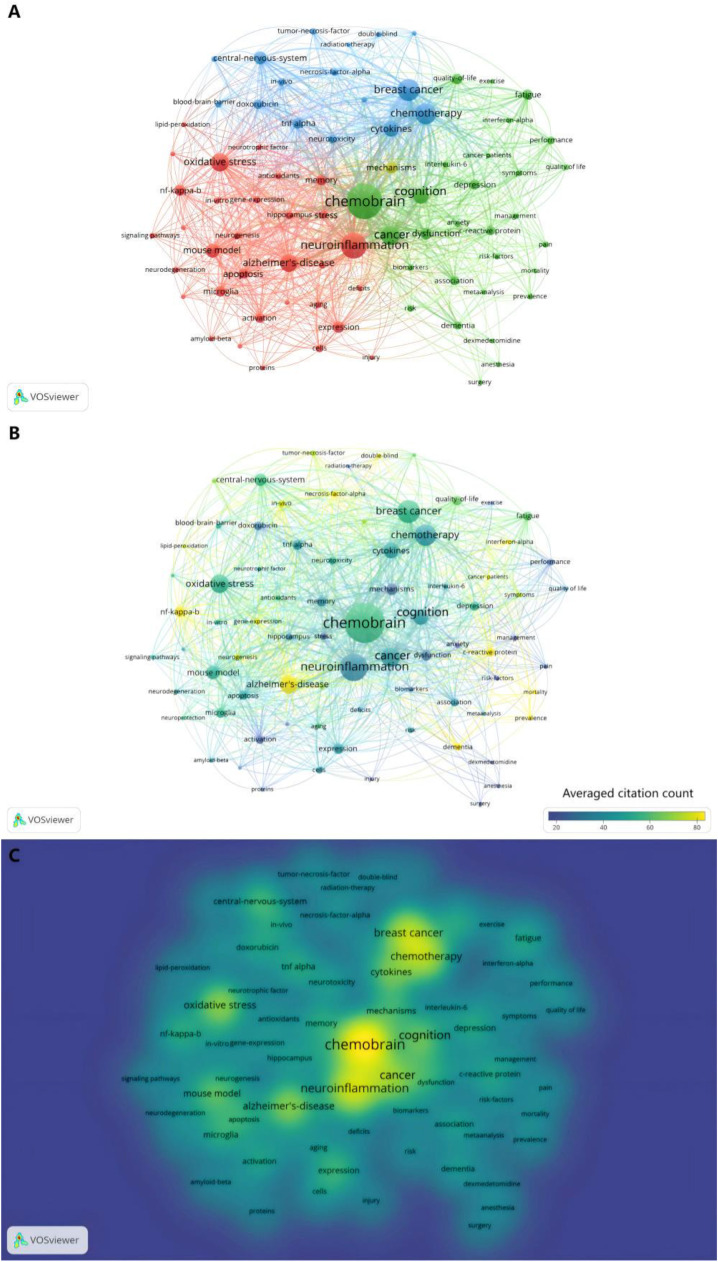
**Term visualization for 1994‒2023.** (A) Term network visualization for 1994‒2023.This map shows the visualization of 78 terms occurred at least 15 times in publications from 1994 to 2023. Each circle in map represents a term. The size of the circle is proportional to the occurrence of the term (the bigger the circle, the more frequency of occurrence). The thickness of the connecting lines is proportional to the link strength of terms (the thicker the line, the more correlation of terms). (B) Term overlay visualization for 1994‒2023. Colors range from blue to green to yellow. The more often the term is referenced, the closer the color of the dot is to yellow. The other way around, the less often the term is referenced, the closer the color of the dot is to blue. (C) Term density visualization for 1994‒2023. Each point in the density visualization has a color that indicates the density of terms at that point. Colors range from blue to green to yellow. The larger the number of terms in the neighborhood of a point and the higher the weights of the neighboring terms, the closer the color of the point is to yellow. The other way around, the smaller the number of terms in the neighborhood of a point and the lower the weights of the neighboring terms, the closer the color of the point is to blue.

The most recent period (2014‒2023) reflects a mature and diversified research arena. Frequently occurring terms now form a complex network including “chemobrain”, “neuroinflammation”, “oxidative stress”, and ‒ for the first time ‒ “blood-brain barrier”, indicating a sophisticated focus on central nervous system penetration and multifaceted damage mechanisms. The density visualization (Fig. 3C) clearly shows the overwhelming dominance of these terms, confirming their central role in the current research paradigm. Furthermore, the high citation impact of terms like “dementia” and “Alzheimer's disease” signals a growing interest in exploring the potential connections between chemotherapy-induced cognitive decline and neurodegenerative diseases (Fig. 2C). This shift implies a significant scientific progression: the field is now actively situating chemobrain within the broader context of neurocognitive disorders, potentially enabling knowledge transfer from more established neurodegenerative research. The overarching analysis of the entire 30-year period consolidates “chemobrain”, “neuroinflammation”, “breast cancer”, and “chemotherapy” as the enduring core of this research domain (Fig. 3A–C).

### Geographical and institutional contributions

**Fig. 4:**
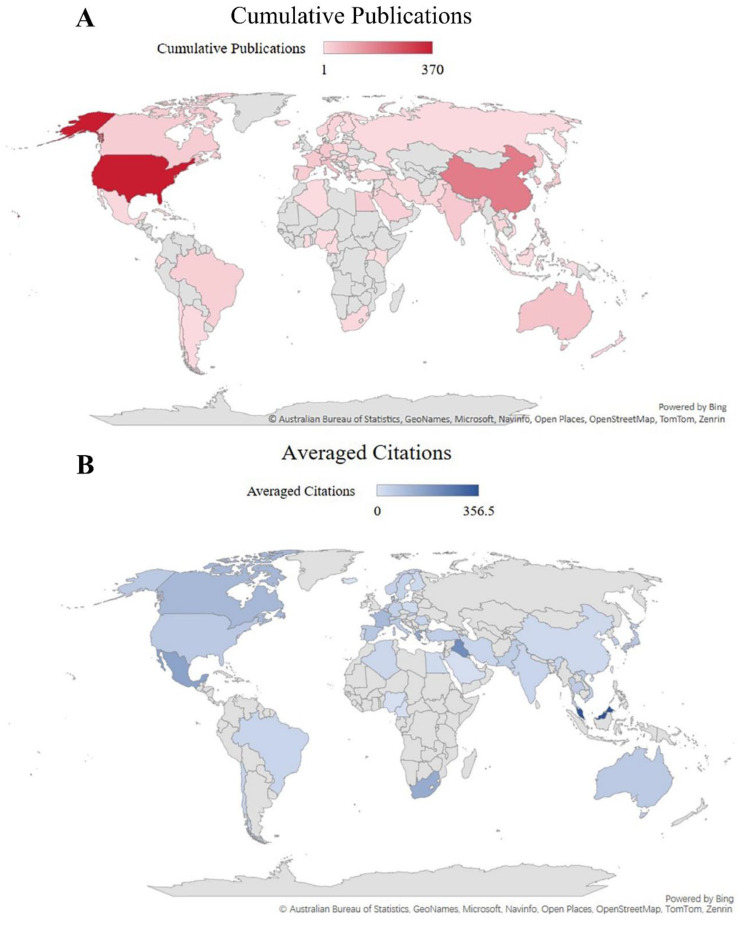
**World maps depicting the geographical distribution of neuroinflammation and chemobrain-related publications, 1994‒2023.** Authors may be from multiple countries, indicating international collaboration. (A) Cumulative publication counts per country. Please refer to the color scale. (B) Averaged citations per article per country. Please refer to the color scale.

The geographical distribution of research output reveals a dynamic and shifting landscape of global leadership. The United States established itself as the dominant force from the outset, contributing over half of all publications during 1994‒2003 and maintaining a commanding lead with around 60 % of the output in the following decade (2004‒2013). The world map depicting cumulative publications (Fig. 4A) visually reinforces this historical dominance. While the U.S. remained the top contributor in the most recent period (2014‒2023), its global share adjusted to approximately 36 %, largely due to the remarkable ascent of China, which grew to become the second-largest contributor, accounting for about 23 % of publications. However, the map of average citations per article (Fig. 4B) reveals a more nuanced picture, suggesting that while China's quantitative output has surged, the average impact of its publications, as measured by citations, may still be developing compared to some established Western nations. This indicates a potential area for strategic growth in research quality and international influence.

An analysis of research directions shows that the field is primarily anchored in Neurosciences Neurology (25.5 %) and Oncology (17 %), with Pharmacology, Biochemistry, and Immunology representing other major areas (Table 1). While U.S. and Chinese research outputs were strongest in Neurosciences, Australian and Italian teams demonstrated a comparatively greater focus on Oncology (Table 2). This national productivity is mirrored at the institutional level, where U.S. organizations consistently dominated. Leadership in publication output shifted from the University of Texas System in the first decade, to the University of Kentucky and the UT MD Anderson Cancer Center in the second, and finally to The Ohio State University in the most recent period. The University of California, San Francisco distinguished itself by garnering the highest number of citations from 2014 to 2023. A notable observation is that despite China's formidable rise as a nation, no single Chinese institution ranked among the top-producing organizations in the last decade, suggesting a more distributed research effort across the country rather than concentration within a few elite centers (Tables 3, 4, 5, 6). This presents a strategic consideration for China regarding the potential benefits of building more dominant, world-leading research hubs in this field.

**Table 1:** Top five research directions worldwide 1994–2023.

**Standard Competition Ranking**	**Research Direction**	**Articles (%)**
First	Neurosciences Neurology	235 (25.5 %)
Second	Oncology	156 (17.0 %)
Third	Pharmacology Pharmacy	142 (15.4 %)
Fourth	Biochemistry Molecular Biology	109 (11.8 %)
Fifth	Immunology	76 (8.2 %)

**Table 2:** Top five research directions of five most productive countries 1994–2023.

**Standard Competition Ranking**	**Country**	**No.**	**Research Directions**	**Articles**	**% of All Articles in the Country**	**% of All Articles in the Direction**
First	USA	1	Neurosciences Neurology	94	25.4	40.0
		2	Oncology	87	23.5	55.8
		3	Immunology	41	11.1	53.9
		4	Biochemistry Molecular Biology	39	10.5	35.8
		5	Psychiatry	29	7.8	64.4
Second	China	1	Neurosciences Neurology	48	26.8	20.4
		2	Pharmacology Pharmacy	26	14.5	18.3
		3	Biochemistry Molecular Biology	20	11.2	18.3
		4	Oncology	19	10.6	12.2
		5	Research Experimental Medicine	14	7.8	28.6
Third	Italy	1	Oncology	7	13.2	4.5
		2	Neurosciences Neurology	7	13.2	3.0
		3	Biochemistry Molecular Biology	7	13.2	6.4
		4	Geriatrics Gerontology	6	11.3	13.6
		5	Pharmacology Pharmacy	6	11.3	4.2
Fourth	Australia	1	Oncology	13	28.9	8.3
		2	Neurosciences Neurology	9	20.0	3.8
		3	Pharmacology Pharmacy	5	11.1	3.5
		4	Immunology	4	8.9	5.3
		5	Psychiatry	4	8.9	8.9
Fifth	South Korea	1	Pharmacology Pharmacy	10	24.4	7.0
		2	Neurosciences Neurology	8	19.5	3.4
		3	Biochemistry Molecular Biology	6	14.6	5.5
		4	Nutrition Dietetics	5	12.2	15.2
		5	Chemistry	3	7.3	8.8

**Table 3:** Analysis from 1994 to 2003.

**Top Five Countries in Productivity**				
**Standard Competition Ranking**			**Country**	**Articles (%)**
First			USA	19 (63.3 %)
Second			France	4 (13.3 %)
Third			Scotland	3 (10.0 %)
Fourth			England	2 (6.7 %)
Fourth			Italy	2 (6.7 %)
**Country Distribution of Citing Articles**				
**No.**	**Country**	**Citing Articles**	**% of All Countries**	**Citations Per Publication**^a^
1	USA	3724	79.4	196
2	France	691	14.7	172.75
3	Scotland	521	11.1	173.67
4	England	137	2.9	68.5
5	Italy	96	2.0	48
All countries		4691	‒	156.37
**Top Three Productive Authors**				
**Standard Competition Ranking**		**Author**	**Affiliation**	**Publications**
First		Meyers CA	Planet Youth ehf, Reykjavik, Iceland	6
Second		Capuron L	Universite de Bordeaux, Bordeaux, France	3
Second		Dantzer R	UTMD Anderson Cancer Center, Houston, TX, USA	3
**Author Analysis of Citing Articles**				
**No.**	**Author**	**Citing Articles**	**% of All Authors**	**Citations Per Publication**
1	Miller AH	954	19.2	477
2	Meyers CA	923	18.6	153.8
3	Capuron L	903	18.2	301
4	Dantzer R	683	13.7	227.7
All authors		4961	‒	156.37
**Productivity Leaders among Journals**				
**Standard Competition Ranking**		**Journal**	**Total (%)**	**IF (2003)**
First		*Cancer*	4 (13 %)	4.017
Second		*Journal of the International Neuropsychological Society*	2 (7 %)	2.304
Second		*Seminars in Oncology*	2 (7 %)	4.733
**Citing Articles by Journal**				
**No.**	**Journal**	**Citing Articles**	**% of All Journals**	**Citations Per Publication**
1	*Neuropsychopharmacology*	587	11.8	587
2	*Cancer*	543	10.9	135.8
3	*Nature Reviews Drug Discovery*	540	10.9	540
4	*Journal of the International Neuropsychological Society*	414	8.3	207
5	*Prostaglandins Leukotrienes and Essential Fatty Acids*	405	8.2	405
Total of 1‒5		2489	50.2	276.6
All Journals		4961	‒	156.37
**Ranking the Top Productive Organizations**				
**Standard Competition Ranking**		**Organizations**		**Articles ( %)**
First		University of Texas System		6 (20.0 %)
Second		Emory University		4 (13.3 %)
**Institutional Analysis of Citing Articles**				
**No.**	**Institution**	**Citing Articles**	**% of All Institutions**	**Citations Per Publication**
1	Emory University	1270	25.6	317.5
2	University of Texas System	1057	21.3	176.2
3	Merck & Co Inc	540	10.9	540
3	Pharmacia	540	10.9	540
5	University of Aberdeen	440	8.9	220
All Institutions		4961	‒	156.37

a Equals citing articles divided by the number of publications from each respective country.

**Table 4:** Analysis from 2004 to 2013.

**Top Five Countries in Productivity**					
**Standard Competition Ranking**			**Country**	**Articles (%)**	
First			USA	79 (59.0 %)	
Second			France	13 (9.7 %)	
Third			Italy	10 (7.5 %)	
Fourth			Canada	8 (6.0 %)	
Fifth			Germany	7 (5.2 %)	
**Country Distribution of Citing Articles**					
**No.**	**Country**	**Citing Articles**	**% of All Countries**	**Citations Per Publication**^a^	
1	USA	9378	66.2	120.2	
2	France	2054	14.5	158	
3	Japan	1230	8.7	246	
4	Canada	1218	8.6	152.3	
5	Australia	1056	7.4	176	
All countries		14,175	‒	105.78	
**Top Four Productive Authors**					
**Standard Competition Ranking**			**Author**	**Affiliation**	**Publications**
First			Sultana R	University of Texas Southwestern Medical Center Dallas, Dallas, TX, USA	7
Second			Butterfield DA	University of Kentucky, Lexington, KY, USA	6
Third			Vore M	University of Kentucky, Lexington, KY, USA	5
Fourth			Robbins ME	Stanley Manne Children's Research Institute, Chicago, IL, USA	4
**Author Analysis of Citing Articles**					
**No.**	**Author**	**Citing Articles**	**% of All Authors**	**Citations Per Publication**	
1	Sultana R	719	5.1	102.7	
2	Meyers CA	664	4.7	221.3	
3	Vore M	640	4.5	128	
4	Butterfield DA	593	4.2	98.8	
5	Bernstein LJ	561	4	280.5	
All authors		14,175	‒	105.78	
**Productivity Leaders among Journals**					
**Standard Competition Ranking**			**Journal**	**Total (%)**	**IF (2013)**
First			*Free Radical Biology and Medicine*	5 (3.7 %)	5.71
Second			*Brain Behavior and Immunity*	3 (2.2 %)	6.128
Second			*PLoS One*	3 (2.2 %)	3.534
Second			*Journal of Clinical Oncology*	3 (2.2 %)	17.96
**Citing Articles by Journal**					
**No.**	**Journal**		**Citing Articles**	**% of All Journals**	**Citations Per Publication**
1	*Journal of Clinical Oncology*		606	4.3	202
2	*Lancet Neurology*		449	3.2	224.5
3	*Brain Behavior and Immunity*		391	2.8	130.3
4	*Current Pharmaceutical design*		359	2.5	179.5
5	*PLoS One*		338	2.4	112.7
Total of 1–5			2143	15.1	164.8
All Journals			14,175	‒	105.78
**Ranking the Top Productive Organizations**					
**Standard Competition Ranking**			**Organizations**		**Articles (%)**
First			University of Kentucky		11 (8.2 %)
Second			UTMD Anderson Cancer Center		5 (3.7 %)
Second			University of Rochester		5 (3.7 %)
Third			Emory University		4 (3.0 %)
**Institutional Analysis of Citing Articles**					
**No.**	**Institution**		**Citing Articles**	**% of All Institutions**	**Citations Per Publication**
1	University of Kentucky		916	6.5	83.3
2	University of Rochester		915	6.5	183
3	University of Toronto		852	6	284
4	Emory University		685	4.8	171.3
5	Memorial Sloan Kettering Cancer Center		682	4.8	227.3
All Institutions			14,175	‒	105.78

a Equals citing articles divided by the number of publications from each respective country.

**Table 5:** Analysis from 2014 to 2023.

**Top Five Countries in Productivity**					
**Standard Competition Ranking**			**Country**	**Articles (%)**	
First			USA	272 (35.8 %)	
Second			China	176 (23.2 %)	
Third			Italy	41 (5.4 %)	
Fourth			Australia	38 (5.0 %)	
Fifth			South Korea	36 (4.7 %)	
**Country Distribution of Citing Articles**					
**No.**	**Country**	**Citing Articles**	**% of All Countries**	**Citations Per Publication**^a^	
1	USA	10,605	34.6	39	
2	China	4165	13.6	23.7	
3	Italy	2180	7.1	53.2	
4	Australia	1656	5.4	43.6	
5	South Korea	1609	5.3	44.7	
All countries		30,608	‒	40.33	
**Top Four Productive Authors**					
**Standard Competition Ranking**			**Author**	**Affiliation**	**Publications**
First			Pyter LM	Ohio State University, Columbus, Ohio, USA	11
Second			Acharya MM	University of California Irvine, Irvine, California, USA	8
Second			Chan A	University of California Irvine, Irvine, California, USA	8
Fourth			Alhowail AH	Qassim University, Buraydah, Saudi Arabia	7
Fourth			Butterfield DA	University of Kentucky, Lexington, KY, USA	7
**Author Analysis of Citing Articles**					
**No.**	**Author**	**Citing Articles**	**% of All Authors**	**Citations Per Publication**	
1	Acharya MM	442	1.4	55.3	
2	Limoli CL	383	1.3	63.8	
3	lrwin MR	370	1.2	123.3	
4	Butterfield DA	356	1.2	50.9	
5	Baulch JE	343	1.1	68.6	
All authors		30,608	‒	40.33	
**Productivity Leaders among Journals**					
**Standard Competition Ranking**			**Journal**	**Total (%)**	**IF (2023)**
First			*International Journal of Molecular Sciences*	17 (2.2 %)	4.9
Second			*Brain Behavior and Immunity*	16 (2.1 %)	8.8
Third			*Cancers*	14 (1.8 %)	4.5
Third			*Scientific Reports*	14 (1.8 %)	3.8
Fifth			*Nutrients*	12 (1.6 %)	4.8
**Citing Articles by Journal**					
**No.**	**Journal**		**Citing Articles**	**% of All Journals**	**Citations Per Publication**
1	*Nutrients*		1263	4.1	105.3
2	*Frontiers in Pharmacology*		907	3	82.5
3	*Scientific Reports*		475	1.6	33.9
4	*Brain Behavior and Immunity*		405	1.3	25.3
5	*Molecular Neurobiology*		396	1.3	49.5
Total of 1‒5			3446	11.3	56.5
All Journals			30,608	‒	40.33
**Ranking the Top Productive Organizations**					
**Standard Competition Ranking**			**Organizations**		**Articles (%)**
First			Ohio State University		23 (2.9 %)
Second			University of California, San Francisco		16 (2.0 %)
Third			UTMD Anderson Cancer Center		15 (1.9 %)
Third			University of California, Los Angeles		14 (1.8 %)
Fifth			Ain Shams University		13 (1.6 %)
**Institutional Analysis of Citing Articles**					
**No.**	**Institution**		**Citing Articles**	**% of All Institutions**	**Citations Per Publication**
1	University of California, San Francisco		1457	4.8	91.1
2	University of Toronto		1102	3.6	100.2
3	Stanford University		895	2.9	89.5
4	The University of Sydney		713	2.3	64.8
5	Harvard Medical School		698	2.3	69.8
All Institutions			30,608	‒	40.33

a Equals citing articles divided by the number of publications from each respective country.

**Table 6:** Top Five Countries in Productivity 1994‒2023.

**Standard Competition Ranking**	**Country**	**Articles (%)**
First	USA	370 (40.1 %)
Second	China	179 (19.4 %)
Third	Italy	53 (5.7 %)
Fourth	Australia	45 (4.9 %)
Fifth	South Korea	41 (4.4 %)

### Influential authors and publication venues

The intellectual leadership of the field, as measured by author productivity, has evolved over time. The pioneering phase (1994‒2003) was led by Meyers C.A., while the period of mechanistic expansion (2004‒2013) was defined by Sultana R., who led in both output and citations, reflecting a key period where oxidative stress mechanisms were being firmly linked to chemobrain. In the contemporary era of rapid growth (2014‒2023), Pyter L.M. emerged as the most prolific author, whereas Acharya M.M. accrued the highest number of citations, indicating high-impact contributions, particularly in the area of radiation and chemotherapy-induced neural damage. A consistent trend across these periods is the predominant affiliation of the most productive authors with U.S. institutions, underscoring the country's sustained capacity for nurturing and retaining key opinion leaders (Tables 3‒6).

Concurrently, the preferred publication venues have evolved in tandem with the field's scientific maturation, which in itself is a marker of the field's credibility and integration into established scientific disciplines. Early clinical observations were primarily disseminated in specialized clinical journals such as *Cancer* and *Seminars in Oncology*. As the research emphasis shifted toward understanding biological mechanisms, high-impact journals focusing on fundamental biology like *Free Radical Biology and Medicine* gained prominence. The current landscape is characterized by a diverse array of high-output journals, including the broad-scope *International Journal of Molecular Sciences* and the highly influential, interdisciplinary *Brain, Behavior, and Immunity*. This progression from primarily clinical outlets to a blend of basic science, translational, and high-impact clinical journals illustrates the field's increasing depth, methodological sophistication, and acceptance within the broader scientific community (Tables 3‒6). Publishing in journals with high impact factors and interdisciplinary reach suggests that research on neuroinflammation and chemobrain is now competing for attention and validation within the wider realms of neuroscience and immunology.

## Discussion

### Neuroinflammation and chemotherapy: from bibliometric trends to mechanistic insights

The bibliometric analysis reveals the conceptual evolution of chemobrain research, which has matured from initial phenomenological observations toward a mechanistic dissection of its pathophysiology. This progression beyond mere quantification allows for the synthesis of pivotal neuroinflammatory pathways and the identification of specific molecular mediators linking chemotherapy to cognitive decline.

#### Microglial activation and the pro-inflammatory cytokine cascade

The persistent recurrence and co-occurrence of terms like “TNF” and “microglia” in the term maps (Fig. 2B‒C) underscore the centrality of sustained microglial activation. Chemotherapeutic agents, including those with limited BBB permeability, induce a systemic pro-inflammatory state characterized by elevated circulating cytokines, notably TNF-α and Interleukin-1β (IL-1β).^16^^,^^17^ This peripheral inflammation can be partially attributed to the activation of the pro-inflammatory PI3K/Akt/mTOR axis, which stimulates Nuclear Factor kappa B (NF-κB) activity and subsequent cytokine release.^18^ Upon reaching the brain, these cytokines activate microglia via their cognate receptors, initiating a neuroinflammatory cascade marked by increased CD68 and Ionized calcium-Binding Adapter molecule-1 (IBA-1) expression. This microglial activation, in turn, drives synaptic dysfunction by suppressing Brain-Derived Neurotrophic Factor (BDNF) signaling and promotes neuronal damage through apoptotic and necroptotic pathways.^19^, ^20^, ^21^ Critically, NF-κB acts as a master regulator in this process, amplifying the expression of detrimental cytokines and cementing a self-perpetuating cycle of neuroinflammation that disrupts hippocampal neurogenesis and cognitive function, thereby underpinning chemobrain.^22^

#### Blood-brain barrier disruption and central infiltration

The emergence of “blood-brain barrier” (Fig. 2C) as a significant keyword is substantiated by findings that chemotherapeutics like cisplatin and methotrexate induce persistent BBB disruption, as demonstrated by increased permeability to tracers of varying molecular weights months post-treatment.^23^ This breach is mechanistically driven by chemotherapy-induced DNA damage, which triggers p16 pathway activation and cellular senescence in cerebromicrovascular endothelial cells and microglia.^24^ Senescent cells adopt a Senescence-Associated Secretory Phenotype (SASP), characterized by the release of pro-inflammatory cytokines and Matrix Metalloproteinases (MMPs), which collectively degrade tight junction protein. The consequent structural compromise of the BBB allows for the paracellular influx of neurotoxic molecules and inflammatory mediators into the CNS parenchyma. This infiltration, coupled with direct SASP signaling from senescent microglia, sustains a state of chronic neuroinflammation and oxidative stress, ultimately leading to synaptic dysfunction, white matter damage, and the cognitive deficits that define chemobrain.^23^, ^24^, ^25^

#### Oxidative stress, lipid peroxidation, and mitochondrial dysfunction

The robust co-occurrence of “oxidative stress” and “lipid-peroxidation” in recent literature underscores the contribution of direct oxidative damage. Anthracycline-based agents, among others, catalyze the excessive production of Reactive Oxygen Species (ROS), driving lipid peroxidation. A critical target of this process is Apolipoprotein A1 (ApoA1), which undergoes oxidative modification by lipid-derived aldehydes such as 4-Hydroxy-2-trans-Nonenal (HNE).^26^^,^^27^ This modified ApoA1 is associated with elevated systemic TNF-α, which propagates oxidative stress into the CNS.^28^ Within neurons, ROS overwhelm mitochondrial defenses, leading to nitrative inactivation of the antioxidant enzyme Manganese Superoxide Dismutase (MnSOD).^16^ The consequent mitochondrial failure impairs energy metabolism, promotes macromolecular damage, and initiates apoptotic signaling, culminating in neuronal loss and cognitive deficits.

#### The gut-brain axis and systemic immune communication

The gut-brain axis serves as a significant indirect pathway through which systemic chemotherapy may provoke central neuroinflammation and cognitive decline. Chemotherapy-induced intestinal dysbiosis and mucosal injury facilitate the translocation of bacterial products, notably Lipopolysaccharide (LPS), into the systemic circulation. Upon entry, LPS acts as a potent pathogen-associated molecular pattern, binding to Toll-Like Receptor-4 (TLR4) on innate immune cells and triggering a robust peripheral inflammatory response characterized by the release of pro-inflammatory cytokines such as TNF-α and IL-6.^29^ These circulating inflammatory mediators can subsequently access the central nervous system, where they promote a neuroinflammatory state. This state is perpetuated by activated astrocytes and sustained cytokine signaling, which collectively contribute to neuronal dysfunction and apoptosis. The resulting impairments in synaptic plasticity and hippocampal-dependent memory processes represent a plausible mechanistic basis for the cognitive deficits observed in CICI.^30^

In summary, the bibliometric mapping corroborates a multi-hit pathogenic model for chemobrain, wherein chemotherapy initiates a cascade of peripheral events ‒ including gut dysbiosis, systemic inflammation, and oxidative stress ‒ that converge to instigate central neuroinflammation. The core mechanistic themes elucidated herein involve the pivotal roles of NF-κB-driven cytokine release, microglial activation, BBB disruption, and mitochondrial dysfunction, which collectively disrupt synaptic plasticity and neuronal homeostasis.

### Translational implications

#### Potential biomarkers for clinical application

The evolving landscape of CICI research has identified a range of blood-based biomarkers with potential clinical relevance, including immune, genetic, neuroendocrine, and other circulating factors.

Among immune-related biomarkers, cytokines have been the most extensively studied. Elevated levels of pro-inflammatory cytokines, particularly IL-6 and TNF-α, have shown the most consistent associations with both subjective and objective measures of cognitive impairment across multiple studies.^31^^,^^32^ Other cytokines, including IL-1β, IL-2, IL-4, and IL-8, have also demonstrated significant, though less consistent, relationships with CICI.^33^ Beyond cytokines, C-Reactive Protein (CRP) has emerged as a promising systemic inflammatory marker, with higher levels correlating with poorer cognitive performance in several studies.^34^ Additionally, cellular immune markers such as white blood cell counts, neutrophil counts, and CD4^+^/CD8^+^ *T*-cell ratios have been linked to global cognitive performance, suggesting their potential utility as indicators of neuroinflammatory states associated with chemotherapy.^35^^,^^36^

Genetic biomarkers represent another promising avenue for CICI prediction and stratification. The APOE ε4 allele, while showing inconsistent associations across studies, has been identified as a potential modifier of cognitive outcomes in specific patient subgroups and treatment contexts.^37^ Variations in Brain-Derived Neurotrophic Factor (BDNF) genes, particularly the rs6265 polymorphism, have demonstrated protective effects against CICI development.^38^ Furthermore, single nucleotide polymorphisms in cytokine genes, such as IL1R1, IL6, TNF), Catechol-O-Methyltransferase (COMT), and DNA repair genes like ERCC5 have shown significant associations with cognitive trajectories, highlighting the potential of genetic profiling for risk stratification.^39^, ^40^, ^41^, ^42^, ^43^

Other biomarker categories have yielded additional insights. Circulating BDNF levels have shown inverse relationships with CICI severity, with lower levels associated with greater cognitive impairment.^44^ Neurofilament proteins, while not consistently associated with CICI in initial studies, warrant further investigation given their established role as markers of axonal damage in other neurological conditions.^45^ Emerging biomarkers such as mitochondrial DNA content, telomere length, DNA methylation patterns, and total RNA gene expression profiles have shown preliminary associations with cognitive outcomes, suggesting novel pathways for mechanistic exploration and biomarker development.

The integration of blood-based biomarkers, including inflammatory markers, genetic susceptibility profiles, and neurotrophic factor, into multimodal panels represents a promising translational approach that may significantly improve predictive accuracy and enable early identification of at-risk patients. Future validation in large prospective cohorts and standardization of measurement protocols are essential next steps. Furthermore, incorporating advanced neuroimaging modalities, particularly functional MRI for assessing functional connectivity and structural MRI for evaluating hippocampal volume and white matter integrity, could provide complementary non-invasive measures of central nervous system alterations, thereby establishing a more comprehensive framework for understanding and monitoring chemotherapy-related cognitive impairment.^46^^,^^47^

#### Therapeutic strategies targeting neuroinflammation

The bibliometric analysis reveals a growing research focus on interventions targeting neuroinflammatory pathways, suggesting promising therapeutic avenues. The identification of specific agents and pathways in the literature, such as “curcumin”, “vitamin E”, and mechanisms involving “microglia” and “NF-Κb”, points to potential pharmacological strategies. Preclinical studies have demonstrated that compounds like curcumin, with its anti-inflammatory and antioxidant properties, can attenuate neuroinflammation and improve cognitive outcomes in animal models of chemobrain.^48^ Similarly, the potential of other agents, including minocycline (an inhibitor of microglial activation), N-acetylcystein, and various natural products, is supported by their association with key mechanistic terms.^49^ These findings advocate for the translation of such candidates into well-designed clinical trials to evaluate their efficacy in preventing or treating cognitive impairment in cancer patients. Beyond pharmacological approaches, the analysis also implies the relevance of non-pharmacological interventions. Terms related to “exercise”, “diet”, and “cognitive training”, though perhaps less explicitly frequent in keyword analyses, align with broader research trends suggesting that lifestyle interventions can modulate inflammation and support cognitive health. Combining targeted anti-neuroinflammatory pharmacotherapy with structured non-pharmacological interventions, such as aerobic exercise and cognitive rehabilitation, may represent a comprehensive and effective strategy.^50^ Future research should prioritize clinical trials that integrate these multimodal approaches, focusing on patient populations most vulnerable to chemobrain, to establish robust, clinically actionable therapeutic protocols.

### Limitation and improvement

The authors employed bibliometrics to analyze development and trends in the field of neuroinflammation and chemotherapy. While bibliometric analysis offers a relatively objective and comprehensive approach, it is subject to several common limitations. In this study, only the Web of Science Core Collection (WoSCC) was utilized for data retrieval. Although WoSCC is widely regarded as a leading and authoritative database in scientific research, its exclusive use may limit the comprehensiveness of the dataset, as other major databases ‒ such as PubMed, Scopus, and Embase ‒ contain substantial relevant literature not indexed in WoSCC. Therefore, the omission of these sources represents a notable constraint in the scope of the present analysis. Additionally, as is typical in bibliometric studies, no formal quality assessment of the included publications was conducted, meaning that articles of varying quality were treated equally ‒ a factor that may influence the interpretation of results. Looking forward, future bibliometric studies in this field could be enhanced by incorporating multiple databases to achieve a more comprehensive coverage of the literature. Integrating data from sources such as PubMed, Scopus, and Embase alongside WoSCC would provide a broader and more representative sample of the research landscape, thereby strengthening the robustness and generalizability of the findings.

## Conclusion and future directions

In summary, this bibliometric analysis maps the evolving intellectual landscape of chemotherapy-related cognitive impairment and highlights the growing consensus around neuroinflammation as a pivotal mechanistic hypothesis. The field has matured from early phenomenological reports to increasingly nuanced explorations of molecular pathways, with prominent research themes encompassing microglial activation, blood-brain barrier integrity, oxidative stress, and gut–brain axis communication. It is crucial to recognize, however, that the associations and trends revealed through bibliometric methods reflect patterns in terminology and scholarly focus ‒ not direct biological evidence. Therefore, these findings should be regarded as indicators of evolving research priorities and conceptual shifts, rather than as validation of pathophysiological mechanisms.

Moving forward, future research should prioritize hypothesis-driven investigations to empirically validate the neuroinflammatory pathways highlighted in this analysis. Key research avenues should aim to delineate the temporal dynamics of microglial activation, cytokine-mediated signaling, and blood-brain barrier disruption in the aftermath of chemotherapy. The integration of multi-omics methodologies with longitudinal neuroimaging and systematic biomarker profiling will be crucial for elucidating these mechanistic links. In parallel, computational biology offers a promising strategy for the systematic screening of microbiome-derived metabolites and plant-based phytochemicals, serving as a novel source of potential neuroprotective or anti-cancer agents, as evidenced by recent in silico studies. Ultimately, well-designed clinical trials evaluating both pharmacological and non-pharmacological anti-neuroinflammatory interventions will be essential to translate these preclinical and in silico insights into effective cognitive preservation strategies for cancer patients.

By addressing these research gaps, subsequent investigations can rigorously evaluate the role of neuroinflammation in chemobrain and inform the development of targeted interventions to maintain cognitive health in cancer survivors.

## Informed consent

Not applicable.

## Ethics committee information

Not applicable.

## Funding

This work was supported by the 10.13039/501100001809National Natural Science Foundation of China (n° 82271373), and 10.13039/501100005089Beijing Municipal Natural Science Foundation (n 7232075).

## Data availability

The datasets generated and/or analyzed during the current study are available from the corresponding author upon reasonable request.

## Declaration of competing interest

The authors declare that the research was conducted in the absence of any commercial or financial relationships that could be construed as a potential conflict of interest.
